# Pterygium surgery using the principle of contact inhibition: results of 13 years’ experience

**DOI:** 10.1007/s00417-016-3558-9

**Published:** 2016-11-28

**Authors:** Tsutomu Hara, Takako Hashimoto, Takeshi Hara

**Affiliations:** Hara Eye Hospital, 1-1-11 Nishi, Utsunomiya, 320-0861 Japan

**Keywords:** Contact inhibition, Conjunctiva, Cornea, Mitomycin, Pterygium

## Abstract

**Purpose:**

To report a technique to prevent pterygium recurrence using the principle of contact inhibition.

**Methods:**

Two hundred and fifteen patients (232 eyes; average age, 64.1 years) with primary pterygia who underwent pterygium surgery at the Hara Eye Hospital between 1999 and 2012. We retrospectively evaluated the patients who underwent the following procedure to prevent pterygium recurrence. The surface conjunctiva on the pterygium body was not removed. After removing the pterygium body, by placing a narrow pedicle autoconjunctival flap along the corneal limbus and tying it tightly to the front area of the residual conjunctiva, there is no room for the active residual tissue to proliferate, thus preventing a recurrence by contact inhibition. The key factor is conjunctival suturing, which establishes face-to-face contact of both areas of cut conjunctival tissue. Mitomycin C is applied locally for 3 min intraoperatively and 5 days postoperatively. The main outcome measure was the prevention of pterygium recurrence using this technique.

**Results:**

By the end of the average follow-up of 5 years 4 months, three eyes (1.3%) had a recurrence. Among the 232 eyes, 23 eyes had large pterygia extending to the pupillary area. Using the surgical technique, there were no recurrences. No specific characteristic of the recurrence was found in association with the eye, sex, and preoperative grade.

**Conclusions:**

This surgery has three relevant features: (1) reconfirmation of the effect of contact inhibition, (2) the anatomic structure of the conjunctival sac scarcely changes postoperatively, because the surface conjunctiva of the pterygium body is not removed, and (3) a low recurrence rate.

**Electronic supplementary material:**

The online version of this article (doi:10.1007/s00417-016-3558-9) contains supplementary material, which is available to authorized users.

## Introduction

In 1994, Hara and associates reported a new surgical technique for pterygium removal using the principle of contact inhibition [1]. Contact inhibition is a critical mechanism that regulates in-vitro and in-vivo cellular proliferation in a multicellular component [2–4]. When a single cell is cultured, it continues to grow; however, when the culture is filled with cells, the cellular growth stops. The mechanism is the same when two different cellular groups are cultured in one plate. When the front lines of both groups meet, the growth of each stops.

Many methods have been used to treat pterygia with differing results. In 2003, Hirst summarized the previous surgeries; his concept remains valid today [5], In 2013, Kaufman et al. reported a new summary of cases from 2004 to 2011 in a report by the American Academy of Ophthalmology [6]. Although there was no big change, the effect of mitomycin C (MMC) is emphasized more in the latter. Generally, the bared sclera technique was considered obsolete and the technique in which the bared sclera was covered by conjunctiva became the standard. There were two types of coverings, one covering only the defect and one to protect the limbal corneal stem cells. An amnionic membrane graft was used in cases with more progressed disease. But no one commented on our 1994 report on the use of the pedicle conjunctival flap with the hypothesis of contact inhibition. Although it is a phenomenon in cell biology and we had no fundamental proof, we speculated that the concept might be useful in pterygium surgery.

Many reports of pterygium recurrence have been published. With the bared scleral technique, the recurrence rates have ranged from 11 to 61% [7–10]. When the conjunctival defect was covered, the recurrence rates ranged from 0 to 10% [9, 11–17]. Limbal conjunctival transplantation has been associated with recurrence rates ranging from 0 to 7.4% [15, 16, 18–20]. When a rotated conjunctival flap was transplanted, 3% of eyes had a recurrence [21]; with corneal limbal transplantation of the corneal stem cells, 4.75% [22]; and with amnionic membrane transplantation, 10.9% [15] and 25.0% [23]. In 2011, Hirst reported that no pterygia recurred using his technique, and the results of pterygium surgery have to be defined by how the normal conjunctival condition is retained [24].

However, with so many surgical procedures and different results, the problem of recurrence is clearly unresolved. Recognition of a universal surgical principle to apply to most pterygium surgeries is required, which brought us to the improvement of our method in 1994.

In our previous report of contact inhibition, the recurrence rate was 10.7% [1], which was still too high. However, during the current 20-year trial, the key surgical factors were clarified and we obtained markedly improved results. We speculated that the principle of contact inhibition seems to be the fundamental factor in most pterygium surgeries. Many surgeons believe that the technique used to treat primary pterygium is insufficient for secondary pterygia. However, secondary pterygia occur when the first pterygium surgery fails. Therefore, establishing a successful technique to treat primary pterygia is important.

## Material and methods

Two hundred and fifteen patients (232 eyes) (74 men, 141 women; average age, 64.1 ± 12.0 years; range, 21–9 3 years) were included who underwent pterygium surgery at the Hara Eye Hospital between January 4, 1999 and December 29, 2012. All patients were consecutive and met three inclusion criteria: one surgeon (Tsutomu Hara) performing all surgeries, the presence of a primary pterygium, and a follow-up period of at least 1 year (average follow-up period, 5 years 4 months ± 3 years 4 months; range, 1 year 0 month s–12 years 6 months). Among all subjects, 23 eyes of 23 patients (10 men, 13 women; average age, 64 years ± 9 years 3 months; range, 49–83 years) had a giant pterygium with a head that extended to the pupillary margin. Among these patients, the average postoperative follow-up was 3 years 9 months ± 2 years 5 months (range, 1 year 0 month –12 years 6 months). The review board of the Hara Eye Hospital approved the study protocol. After the patients received verbal and written explanations of the surgery, each provided informed consent.

## Surgical methods

To obtain excellent results with contact inhibition, the surgical technique must be precisely performed. Sutures must be placed to induce contact inhibition. All surgeries were performed under a microscope. After instilling a drop of oxybuprocaine hydrochloride (Benoxil, Santen Pharmaceutical, Osaka, Japan), a lid speculum was put in place. Local anesthesia was induced with a subconjunctival injection of 0.2 ml of 2% lidocaine hydrochloride (Xylocaine, AstraZeneca, Osaka, Japan) including 0.0005% adrenaline (Bosmin, Daiichi Sankyo, Tokyo, Japan).

**Fig. 1:**
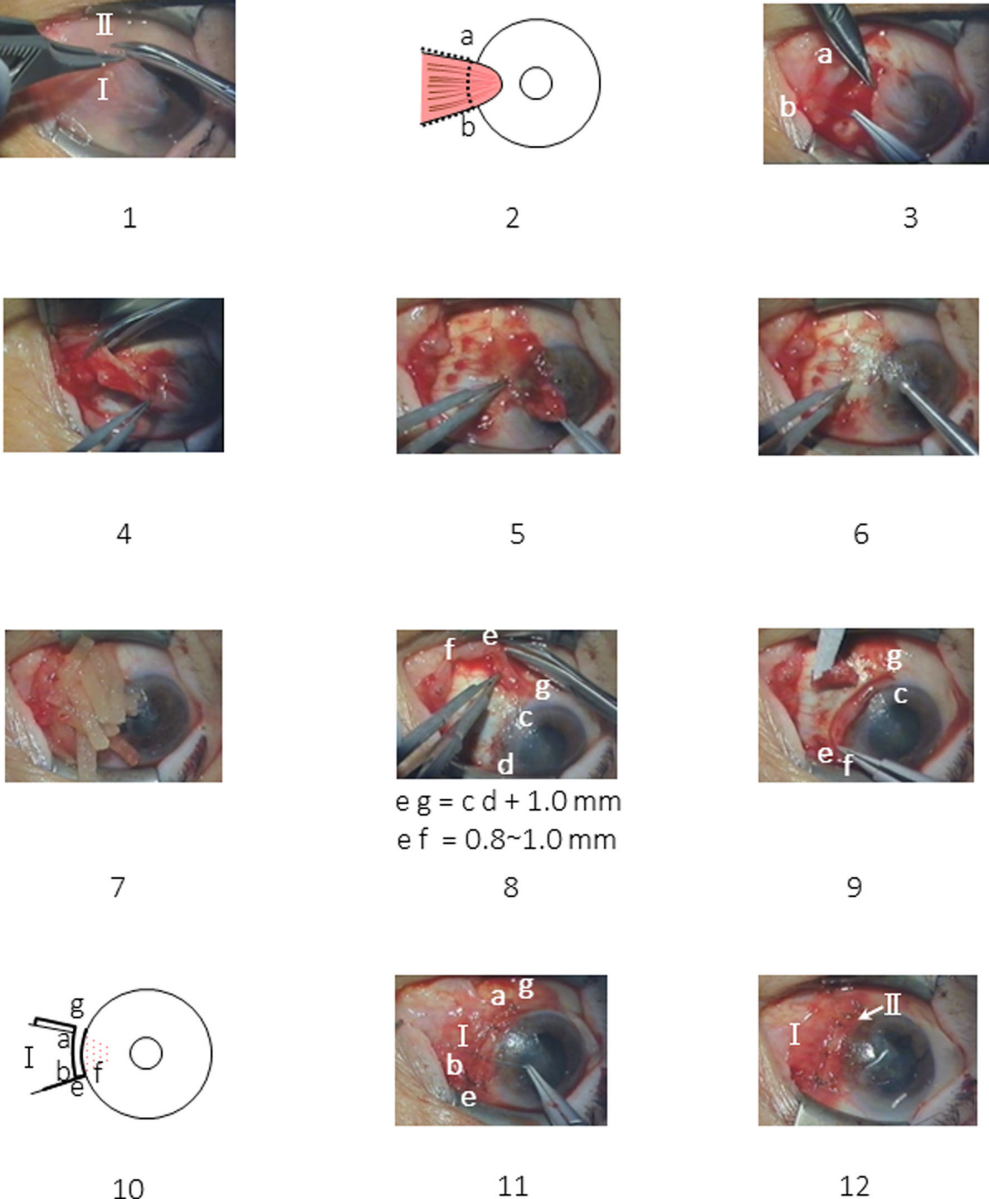
The surgical process. (surgeon’s view of the left eye.) *1* The initial surgical plan. The dotted line indicates the planned incision site of the surface conjunctiva. I, Pterygium body. II, Pedicle conjunctival flap. *2* Surgical process. Incision of the surface conjunctiva. *3* Knots are placed at both corners of the conjunctival flap (*a*, *b*). The surface conjunctiva is turned over. *4* The pterygium body is resected. *5* Gross removal of the pterygium head with peeling or cut. 6, The corneal surface is smoothed with an electric drill. *7* A sponge is soaked with mitomycin C followed by washing with Ringer’s solution. *8* Creation of a pedicle conjunctival flap. Head of the pedicle conjunctival flap (*e*, *f*). Root of the pedicle flap (*c*, *g*). *9* The pedicle flap is rotated 90 degrees to the corneal limbus. The head of the pedicle flap (*e*, *f*) is sutured. A needle is passed in the following order: conjunctival flap, episclera, and conjunctiva. *10* The surface conjunctiva (I) is returned to its original position. *11* Suturing of the surface conjunctiva of the pterygium body. Suture a-g, b-e. Additional sutures at the line of contact inhibition (*e*–*g* and *a*–*b*). Two to four sutures usually are placed. *12* Final sutures are placed for the superior and inferior sides of *I*. Two to three sutures usually are required for each side

**Fig. 2:**
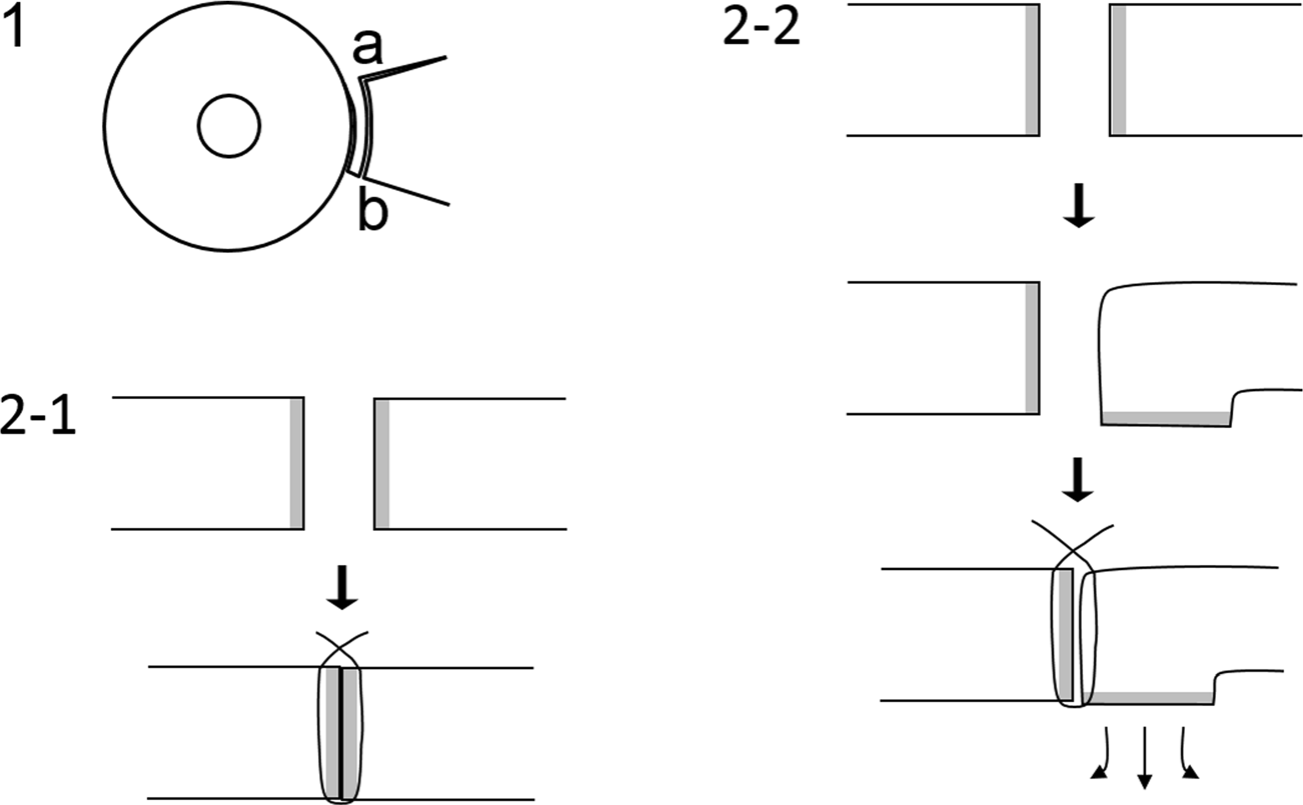
Face-to-face suturing. *1* Overview. *2–1* Side view of correct face-to-face suturing to induce contact inhibition preventing proliferation. *2–2* Side view of incorrect suturing. If a correct face-to-face suturing is not performed, proliferation occurs

**Fig. 3:**
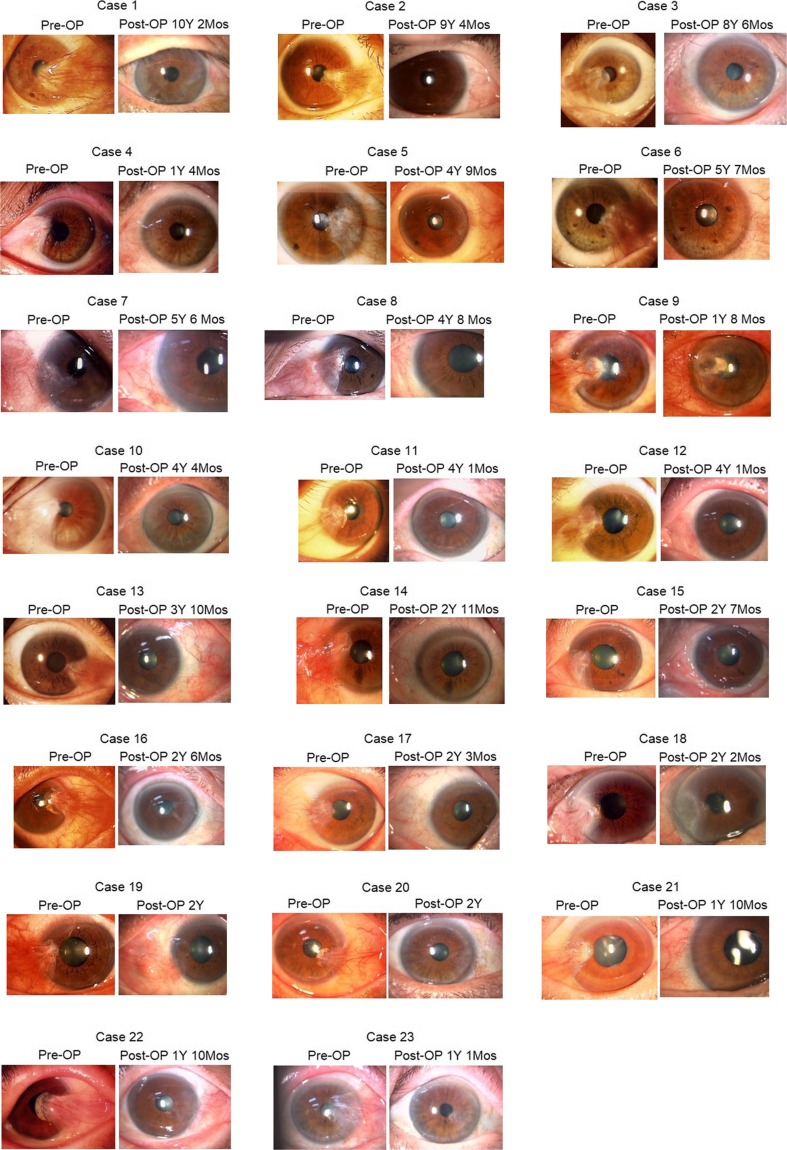
Twenty-three cases of giant pterygium that extend to the pupillary area. *Pre-OP*; preoperative finding, *Post-OP*; postoperative finding, *Y* = years; *Mos* = months

Figure 1 shows the surgical steps. Figure 1-1 (surgeon’s view) shows the basic surgical concept. The surface conjunctiva is cut slightly from the corneal limbus (a-b) and both the superior and inferior edges of the pterygium body (Fig. 1-2). A conjunctival flap is created (Fig. 1-3). Sutures are placed to mark each corner of the square conjunctival flap (a, b). After the conjunctival cut, the thin conjunctiva generally shrinks and the front portion shrinks inward. Rapid identification of the exact cut edge of the front conjunctival flap becomes difficult. To obtain contact inhibition, exact face-to-face suturing of the dissected tissue edges is indispensable. The marking sutures are important, especially during the initial learning period.

The pterygium body is resected maximally (Fig. 1-4). The head of the pterygium in the cornea is removed grossly with scissors or evulsion with forceps (Fig. 1-5). The residual tissue in the cornea then is removed using an electric trephine. The rotation speed should be as fast as possible and the strength of grinding is determined by the grade of the drill touching the cornea (Fig. 1-6).

A sponge soaked with 0.04% MMC (Kyowa Hakko Kirin, Tokyo, Japan) is placed on the scleral surface and inner surface of the conjunctival flap for 3 min (Fig. 1-7). The tissue is then rinsed with 150 ml of lactated Ringer’s solution.

The pedicle autoconjunctival flap usually is created inferiorly considering the future possible surgeries for glaucoma and cataract (Fig. 1-8, f-e-g). Making the pedicle flap (length and width) correctly is important. The cut is performed carefully to avoid pulling the pedicle flap during cutting. If it is pulled excessively, the final width of the pedicle flap becomes narrower, and placing the subsequent sutures for inducing contact inhibition becomes difficult. The length and width of the pedicle flap are important. Even if the flap is short, it can be sutured by pulling; but the width narrows, and suturing to the remaining conjunctiva becomes difficult, resulting in insufficient contact inhibition and possible pterygium recurrence. One millimeter should be added to the width of the pterygium on the corneal limbus, which usually becomes the optimal length of the conjunctival flap, usually 6 to 8 mm. If the flap accidentally becomes free, it can be used as an isolated flap and the surgery can be performed. The recommended width of the pedicle flap (e, f) is usually about 0.8 to 1.0 mm.

When rotating and fixing the pedicle flap, after a 90-degree rotation (Fig. 1-9), the pedicle flap (c-f) is placed along the corneal limbus. The inner side of the pedicle flap (c–f) should be positioned exactly on the curved limbal line at the end of surgery. This inner side (c–f) is not sutured. For exact placement of subsequent sutures, bleeding should be cauterized. Cautery to the corneal limbal sclera, which was used in almost all cases, did not cause noticeable side-effects such as corneal opacification, recurrence, or tissue necrosis. Exact fixation of the pedicle flap is important. The apex of the pedicle flap (e, f) generally is rounded due to the intraoperative contraction. It is important to flatten it by scrubbing with forceps before suturing. At first, both sides of the pedicle flap (e, f) are sutured to the opposite conjunctiva. The thin conjunctiva tends to contract rapidly. If it is sutured only to the conjunctiva, it will soon cause a postoperative wound defect and loss of contact inhibition. Therefore, all sutures must be placed in this order: conjunctival flap, episclera, and opposite conjunctiva (Fig. 1-9).

When suturing the pedicle flap and the surface conjunctiva of the removed pterygium body (body conjunctiva) (Fig. 1-10), the location of the marking sutures must be confirmed and then the cut edges sutured face to face exactly as determined. Seemingly compact suturing observed from above through the operating microscope is not enough. The suturing must be performed as described in this report (Fig. 2).

At the final suturing of the pedicle and body conjunctival flaps (Fig. 1-11), the exact matching of both planes by meticulous suturing induces contact inhibition. This is the most important part of the procedure. When the size and location are prepared correctly, usually three to four sutures are required.

When suturing the upper and lower edges of the body conjunctiva (Fig. 1-12), the body conjunctiva (I in Fig. 1-1) does not extend further, but the conjunctiva on the outside area can be shifted easily. Therefore, the site of needle penetration on the episclera is determined at the body conjunctiva (I in Fig. 1-12). Virgin silk (9–0) is preferable to nylon because of its greater flexibility and reduced postoperative foreign-body sensation. Slight irritation caused by the silk might induce more rapid adherence of both tissues. The usual surgical time is about 40 min. If the inner side of the transplanted conjunctival flap (c, f in Fig. 1-9) is inside the cornea, it should be resected until the corneal limbus appears. While no recurrence develops from this plane, patients may misunderstand it as the recurrence. A video of this surgery is shown in Online Resource.

## Postoperative treatment

The surgical eye is generally covered for 1 week. Sutures are removed 2 weeks postoperatively. From 1 day postoperatively, after lifting the eye cover 0.04% MMC eye drops are instilled 3 times a day for 5 days; 0.5% levofloxacin hydrate eye drops (Cravit Ophthalmic Solution 0.5%, Santen Pharmaceutical) and 0.1% betamethasone sodium phosphate eye drops (Rinderon, Shionogi Pharmaceutical, Osaka, Japan) are used six times daily from 1 day preoperatively for 2 weeks. In addition, 0.1 ml of 0.38% dexamethasone sodium phosphate (Orgadrone, Daiichi Sankyo Co. Ltd.) is injected subconjunctivally three times during the first postoperative week. A vasoconstrictor, 0.5% naphazolin nitrate (Privina Ophthalmic Solution, Novartis Pharma, Tokyo, Japan), is used for 3 months and then discontinued. Some conjunctival hyperemia persists for at least 2 months but usually fades by 3 months postoperatively.

## Results

### Recurrence

If even a small bit of tissue enters the inner side from the corneal limbus during the follow-up, it is considered a recurrence. Of the 232 eyes, three (1.3%) eyes had a recurrence. We found no special relationship between the recurrence and the preoperative appearance and size, age, sex, and grade of the surgical treatment. All recurrences were small and needed no additional surgery at the end of the follow-up period (Table 1). No specific characteristic of the recurrence was found in association with the eye, sex, and preoperative grade.

**Table 1:** Pterygium recurred in three of 232 eyes

Patient	Sex	Eye	Age (years)	Surgical date	Surgery to recurrence (months)	Follow-up (months)
TT	Female	Right	50	2000/3/2	2	47
YY	Male	Right	37	2004/4/9	2	94
WY	Male	Left	54	2007/4/13	21	21

No particular characteristics of the recurrences were identified

The pterygia recurred from the front line of the dissected pterygium body (Fig. 1-2, a-b) in all three eyes; none occurred from either the superior or inferior edges of the body conjunctiva. Among the patients, 23 eyes had a giant pterygium with a head reaching to the pupillary area, none of which recurred. The preoperative and postoperative photographs of the 23 eyes with these giant pterygia are shown to prove the efficacy of the procedure (Fig. 3, cases 1–23).

### Complications

In one eye (no. 9) (Fig. 3-9), a corneal ulcer developed 2 months postoperatively. The patient had a history of herpetic keratitis. The eye healed, leaving a well-covered ulcer. No other complications such as tissue necrosis, restricted ocular movement, cosmetic complaints, or ocular hypertension developed. In some eyes (Fig. 3-16, -18, -22, -23), mild corneal opacification remained as a result of the pterygium depth.

## Discussion

Use of a pedicle conjunctival flap is not new and used widely. What is new is the concept of contact inhibition, although this remains a hypothesis, as the key mechanism for preventing postoperative recurrences of pterygium. We proposed the hypothesis in 1994. The results were not optimal at the time, and we modified some aspects of the previous surgical technique to more effectively facilitate contact inhibition. The results have improved greatly at present. However, some factors still require discussion.

### Key points of the current procedure

To facilitate contact inhibition, full contact must be established at the end of surgery over the entire front line of the excised pterygium body (Fig. 1-2, a-b). We suppose that the chromosomal content of the G0 phase cells, which stop regenerating after sensing tissue contact, should be the same as in the diploid cells. If the cells sense that contact inhibition has ceased, rapid cellular regeneration begins. Thus, tight contact between the tissues at the end of the surgery is important.

Cases in which there was a recurrence, including those in our previous report [1], were thought to have resulted from insufficient contact inhibition, according to our long-term experience. The recurrences resulted from creation of a too short or narrow pedicle flap or overlooking of both the turning down of the front line of the cut conjunctiva or insufficient suturing (Fig. 2). Incomplete contact caused further tissue proliferation.

### New finding of the current procedure

The pedicle flap was intentionally obtained from the area extremely close to the pterygium body (line c-f in Fig. 1-8). Intraoperatively, it is left open toward the center of the cornea without suturing (Fig. 1-9, 1-12), and no tissue regeneration occurred from this line in any cases. The current study proved that in the pterygium and conjunctiva only the line facing the cornea (a–b in Fig. 1-2) has regenerative power. The wide area of the conjunctiva including both the superior and inferior sides had no regenerative power. In addition, the current use of the pedicle flap resulted in almost no change in the anatomic structure of the conjunctival sac.

### Possible factors other than contact inhibition contributing to good results

The first is the effect of MMC as emphasized in the 2013 American Academy of Ophthalmology report [6]. In 1983, we evaluated the use of MMC applied to the postoperative bared sclera. Without MMC, sometimes granuloma occurred, and with high-dose MMC sometimes scleral necrosis occurred [25]. In one eye (case 9) with a giant pterygium, a temporal ulcer developed and healed later, leaving a slight ulcer with a nubecula (Fig. 3-9). Many surgeons have used steroids. Shimazaki and associates reported that steroids must be used for an extended period and they prescribed steroids for 3 months [19]. We used a steroid eye drop for 2 weeks.

### The future

The current study was limited by the absence of a control group and extensive postoperative treatment. The important finding was the low rate of recurrence of 1.3%. The next step is to decrease or eliminate the postoperative medication. We expect that 5 years more are needed before we have definitive results. However, the current new findings, i.e., no need for conjunctival resection of the pterygium body, recurrence site limited to the front cut line of the conjunctiva, the simple surgical technique leaving almost no postoperative changes in the conjunctival structure, and reconfirmation of the effectiveness of the hypothesis of contact inhibition, are useful for future pterygium surgeries.

## Electronic supplementary material

Below is the link to the electronic supplementary material.

An edited 2-minute video of actual surgery. Saved at four times fast play. (MP4 10371 kb)
